# Associations between daily mortality in London and combined oxidant capacity, ozone and nitrogen dioxide

**DOI:** 10.1007/s11869-014-0249-8

**Published:** 2014-03-22

**Authors:** M. L. Williams, R. W. Atkinson, H. R. Anderson, F. J. Kelly

**Affiliations:** 1 MRC-PHE Centre for Environment and Health, Kings College London, Room 4.129 Franklin Wilkins Building, 150 Stamford Street, London, SE1 9NH UK; 2 Public Health Research Institute and MRC-PHE Centre for Environment and Health, St George’s, University of London, London, UK; 3 MRC-PHE Centre for Environment and Health, Kings College London, Franklin Wilkins Building, 150 Stamford Street, London, SE1 9NH UK

**Keywords:** Time series, Mortality, Oxidants, Ozone, Nitrogen dioxide

## Abstract

Both nitrogen dioxide (NO_2_) and ozone (O_3_) are powerful oxidants in ambient air that are intimately linked through atmospheric chemistry and which continuously interchange over very short timescales. Based upon atmospheric chemistry alone, there is a strong, a priori, reason for considering O_3_ and NO_2_ together in epidemiological studies, rather than either of the two pollutants separately in single-pollutant models. This paper compares two approaches to this, using O_x_, defined as O_3_ + NO_2_, as a single metric and also using O_3_ and NO_2_ together in two-pollutant models. We hypothesised that the magnitude of the association between O_x_ and daily mortality would be greater than for NO_2_ and O_3_ individually. Using collocated hourly measurements for O_3_ and NO_2_ in London, from 2000 to 2005, we carried out a time series analysis of daily mortality. We investigated O_3_, NO_2_ and O_x_ individually in single-pollutant Poisson regression models and NO_2_ and O_3_ jointly in two-pollutant models in both all-year and season-specific analyses. We observed larger associations for mean 24-h concentrations of O_x_ (1.30 % increase in mortality per 10 ppb) than for O_3_ (0.87 %) and NO_2_ (0 %) individually. However, when analysed jointly in two-pollutant models, associations for O_3_ (1.54 %) and NO_2_ (1.07 %) were comparable to the O_x_ association. Season-specific analyses broadly followed this pattern irrespective of whether the O_x_ concentrations were driven by O_3_ production (summer) or depletion (winter). This novel approach in air pollution epidemiology captures the simultaneous impact of both oxidants whilst avoiding many of the statistical issues associated with two-pollutant models and potentially simplifies health impact calculations.

**Electronic supplementary material:**

The online version of this article (doi:10.1007/s11869-014-0249-8) contains supplementary material, which is available to authorized users.

## Introduction

Epidemiological time series studies have reported positive associations between outdoor daily concentrations of ozone and nitrogen dioxide and daily counts of health events such as death and admission to hospital (Anderson et al. 2007; WHO 2013). These associations have typically been assessed using regression models incorporating each pollutant individually. It is well known that ozone (O_3_) and nitrogen dioxide (NO_2_), in conjunction with nitric oxide (NO), react together and interchange O_3_ and NO_2_ over a timescale typically of minutes during daytime (Seinfeld and Pandis 2006). The O_3_/NO_x_ system is characterised by the following equations:

1 $$ {\mathrm{O}}_3\kern0.5em +\kern0.5em \mathrm{NO}\kern0.5em =\kern0.5em {\mathrm{NO}}_2\kern0.5em +\kern0.5em {\mathrm{O}}_2 $$

2 $$ {\mathrm{NO}}_2\kern0.5em +\kern0.5em \mathrm{h}v\kern0.5em =\kern0.5em \mathrm{NO}\kern0.5em +\kern0.5em \mathrm{O} $$

3 $$ \mathrm{O}\kern0.5em +\kern0.5em {\mathrm{O}}_2\kern0.5em =\kern0.5em {\mathrm{O}}_3 $$

where h*ν* is a photon causing photolysis of NO_2_. This reaction scheme is closed in the sense that there is no net production or loss of O_3_. At night, or in the absence of sunlight, such as on some winter days, there is no photolysis and reaction (Eq. 2) does not occur. In these situations, if there is sufficient NO, as there usually is in large urban areas, all the ozone is converted to NO_2_ and ozone concentrations are very low or even zero.

There is an important point to note here. These reactions are all relatively fast and constantly interchange O_3_ and NO_2_ very quickly such that, in the absence of other reactions (such as occur in ‘smog’ episodes) and fresh injection of emissions, the sum of O_3_ and NO_2_ is constant, denoted by O_x_ = O_3_ + NO_2_ (Van Egmond and Kesseboom 1985; Clapp and Jenkin 2001). From an atmospheric chemistry point of view, therefore, it would seem appropriate to investigate the combination of O_3_ and NO_2_ together, as the ‘conserved’ pollutant O_x_. An illustration of the atmospheric chemistry and the very close relationship between O_3_ and NO_2_ and O_x_ during the high ozone episode in August 2003 is shown in Fig. S1. This figure, particularly through consideration of the ratio O_3_/O_x_, demonstrates the rapid interchange of O_3_ and NO_2_ over quite short timescales within a day.

Given this dynamic relationship between O_3_ and NO_2_, it is surprising that relatively few time series studies have assessed the health associations of these pollutants jointly using two-pollutant models (Touloumi et al. 1997; Burnett et al. 1998; Gryparis et al. 2004; Simpson et al. 2005; Samoli et al. 2006; HEI 2010). Only a single study of respiratory hospital admissions in Paris has considered O_x_ as a measure of combined oxidative capacity (Chardon et al. 2007). This paper, however, focussed on different metrics for ozone alone. Using collocated measurements for O_3_ and NO_2_ in London, we carried out a time series analysis to investigate the relationships between daily measures of O_3_, NO_2_ and O_x_ and daily all-cause mortality. We hypothesised that despite the dynamic interchange of O_3_ and NO_2_, the magnitude of the association between O_x_ and mortality would be greater than for NO_2_ and O_3_ individually. We also compared the O_x_ associations with the associations for O_3_ and NO_2_ estimated from two-pollutant models.

## Methods

Details of deaths in England and Wales were obtained from the Office for National Statistics. From these records, daily counts of deaths from all non-accidental causes (ICD-10 Chapters A-R) for people resident and dying in London between 1 January 2000 and 31 December 2005 were constructed. Hourly concentrations of NO_2_ and O_3_ were obtained from ten collocated background monitoring stations across London operating during the study period from Defra’s monitoring network and converted from microgram per cubic metre to parts per billion (ppb) using factors of 1.91 and 2 (at 20 C and 1,013 mb) for NO_2_ and O_3_, respectively. Hourly O_x_ concentrations at each site were calculated from the addition of the hourly NO_2_ and O_3_ concentrations. Daily maximum 1-h and mean 24-h (abbreviated to 1-h and 24-h, respectively, hereafter) concentrations of NO_2_, O_3_ and O_x_ were then calculated for each site. Missing values for each pollutant at each site were imputed, in turn, from regression models incorporating daily measures from all monitors and indicators of month and season. London-wide daily 1- and 24-h averages for each pollutant were then calculated by averaging across sites. Daily average temperature and dew point temperature for London (Holborn, a location in central London) were obtained from the British Atmospheric Data Centre website (http://badc.nerc.ac.uk).

We used a Poisson model of daily mortality counts with seasonal patterns modelled as a penalised spline of time with 8 degrees of freedom per year. The model also included natural cubic splines for average daily temperature on the same day and lagged by 1 day (lag 1) and indicator variables for day of week and public holidays. We also stratified our analyses by season (December–February, March–May, June–August and September–November). Each pollutant lagged by 1 day was entered into the models singly (NO_2_, O_3_ and O_x_) and for NO_2_ and O_3_ jointly in two-pollutant models. To assess the shape of the concentration response function for O_3_ and NO_2_ in the all-year data, we explored the shape of the concentration response function for each pollutant individually using natural cubic splines with 3 degrees of freedom. To assess the relationship between mortality and NO_2_ and O_3_ jointly, we modelled the concentration response surface using a bivariate smoothing spline (Wood 2006). As a sensitivity analysis in the all-year model, we incorporated longer lags for temperature (lags 2–6). Relative risks (RR) and 95 % confidence intervals were expressed as percentage changes (100*RR-1) associated with 10 ppb and interquartile range (IQR) increases in pollutant concentrations. The R statistical package was used for all analyses (R, Development Core team 2007).

## Results

Summary statistics for daily mortality counts, O_3_, NO_2_ and O_x_ and temperature, are shown in Table 1. The median number of deaths in London during the study period was 145 per day. One hour concentrations of O_3_ and NO_2_ ranged from 1.7 to 103.9 ppb (interquartile range (IQR) = 13.3 ppb) and from 9.0 to 81.3 ppb (IQR = 12.5 ppb), respectively. One hour O_x_ concentrations were, as expected, higher; range 25 to 123.5 ppb (IQR = 10.6 ppb). Twenty-four hour concentrations of each pollutant had a much lower range reaching maximum values of 55.2, 52.0 and 77.1 ppb for O_3_, NO_2_ and O_x_, respectively. Summary statistics for each pollutant by season are shown in Table S1.

**Table 1:** Summary statistics for daily counts of all-cause mortality, daily mean temperature and daily maximum 1-h and mean 24-h concentrations of ozone (O_3_), nitrogen dioxide (NO_2_) and combined oxidant (O_x_)

Variable	Min^a^	Q1^b^	Med^c^	Q3^d^	Max^f^	IQR^e^
Mortality (n/day)	96	133	145	159	302	26
Temperature (°C)	−0.2	8.4	12.1	16.5	29.3	8.1
Pollutants (ppb)						
1 h						
O_3_	1.7	23.4	29.9	36.6	103.9	13.3
NO_2_	9.0	28	34.2	40.5	81.3	12.5
O_x_	25.0	42.9	47.5	53.5	123.5	10.6
24 h						
O_3_	0.9	10.4	16.5	21.9	55.2	11.5
NO_2_	5.9	16.2	20.4	25.3	52.0	9.2
O_x_	17.4	34.6	38.1	42.3	77.1	7.7

Notes: ^a^ Minimum; ^b^ 25th percentile; ^c^ Median; ^d^ 75th percentile; ^e^ Maximum; ^f^ Interquartile range

**Fig. 1:**
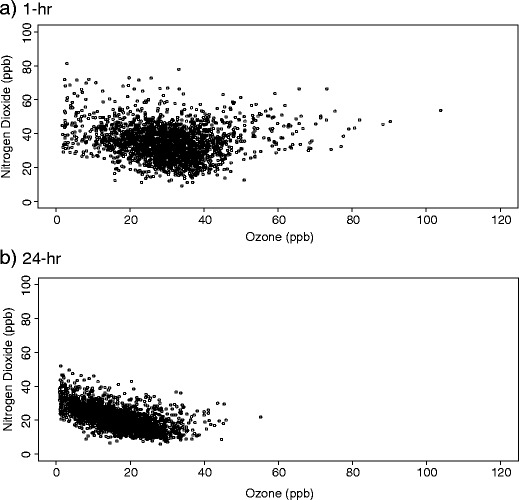
Scatter plot of daily maximum 1-h and mean 24-h nitrogen dioxide and ozone concentrations (ppb) in London between 1 January 2000 and 31 December 2005. **a** 1 h. **b** 24 h

One hour concentrations of NO_2_ and O_3_ were weakly, negatively correlated (Spearman rank correlation coefficient, *r* = −0.1), whereas the 24-h concentrations showed a stronger, negative correlation (Table 2). O_x_ concentrations were more strongly correlated with O_3_ than with NO_2_. The scatter plot of 1-h concentrations of NO_2_ and O_3_ (Fig. 1a) illustrates two different relationships between the pollutants: a negative relationship below about 35 ppb and a positive correlation above. The negative correlation below 35 ppb was also evident in the 24-h measures (Fig. 1b), and the few data points above 35 ppb suggested a positive relationship. Scatter plots and correlation statistics for the pollutants, both 1- and 24-h, by season are given in Figs. S2–S5 and Tables S2–S5. One hour O_3_ and NO_2_ concentrations were negatively correlated in all periods of the year except summer when the pollutants were positively correlated (*r* = 0.51). A similar pattern was observed for 24-h concentrations, although the negative correlations were stronger than for 1-h measures (e.g. −0.71 vs. −0.11 in months March–May) and the positive correlation in the summer months was weaker (0.08 vs. 0.51). During summer months, 1-h O_x_ concentrations were driven by O_3_ (*r* = 0.93); whereas during winter months, they were driven by NO_2_ concentrations (*r* = 0.76). In spring and autumn, even though the overall correlations were negative, at higher ozone levels (above ∼35 ppb), there is an indication in both seasons of a positive correlation in periods of photochemically generated O_3_ as found in the summer months (Figs. S3 and S5).

**Table 2:** Spearman rank correlations coefficients between daily maximum 1-h and mean 24-h concentrations of ozone (O_3_), nitrogen dioxide (NO_2_) and combined oxidant (O_x_)

Pollutant	O_3_	NO_2_	O_X_	O_3_	NO_2_	O_X_
	1 h			24 h		
1 h						
O_3_	1.00					
NO_2_	−0.10	1.00				
O_X_	0.63	0.56	1.00			
24 h						
O_3_	0.87	−0.40	0.39	1.00		
NO_2_	−0.30	0.90	0.43	−0.57	1.00	
O_X_	0.69	0.45	0.91	0.56	0.33	1.00

**Fig. 2:**
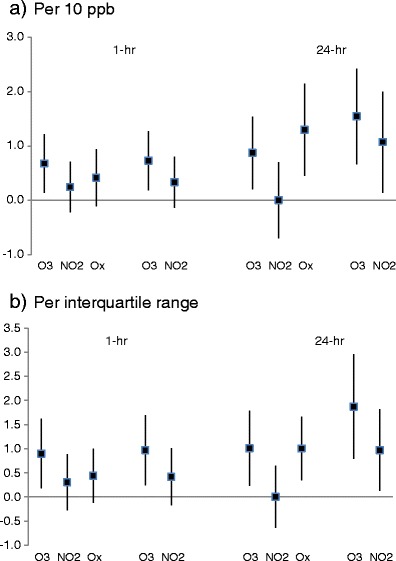
Percentage changes (95 % confidence intervals) in mortality associated with lag 1 day measures of maximum 1-h and mean 24-h concentrations of ozone (O_3_), nitrogen dioxide (NO_2_) and combined oxidant (O_x_) in single-pollutant models and ozone and nitrogen dioxide in two-pollutant models. **a** Per 10 ppb. **b** Per interquartile range

Results from Poisson regression models for mortality and 1- and 24-h O_3_, NO_2_ and O_x_ and two-pollutant models for O_3_ and NO_2_ are shown in Fig. 2, expressed per 10 ppb and per IQR (the corresponding regression estimates and standard errors per parts per billion are tabulated in Table S6). In single-pollutant models, the association (expressed per 10 ppb) for 1-h O_3_ was larger than for NO_2_ (0.68 vs. 0.24 %) with the O_x_ association between the two (0.42 %), although the confidence intervals for each of the three associations overlapped substantially. In two-pollutant models for the 1-h metric, the mutually adjusted associations for O_3_ and NO_2_ were 0.73 and 0.33 %, respectively. The pattern for the 24-h metrics was different however. In single-pollutant models, the association between O_x_ and mortality (1.30 % per 10 ppb) was larger than the single-pollutant model results for O_3_ and NO_2_ (0.87 and 0 %, respectively). The two-pollutant (O_3_ and NO_2_) model associations were 1.54 and 1.07 %, respectively.

Results for individual seasons are illustrated in Figs. S6–S9 with the corresponding regression estimates and standard errors tabulated in Table S6. In summer, and to a lesser extent, during autumn months, O_3_ associations were larger than for NO_2_ and dominated the two-pollutant models. During spring (months March to May), all pollutants were positively associated with all-cause mortality but no associations were observed during winter months. We found that in both the all-year and season-specific analyses, the size of the O_x_ associations were generally in between those for O_3_ and NO_2_ estimated from two-pollutant models whether dominated by O_3_ as during the summer months or NO_2_ as during the spring months. Scaling all associations for the IQRs gave a broadly similar pattern of results.

**Fig. 3:**
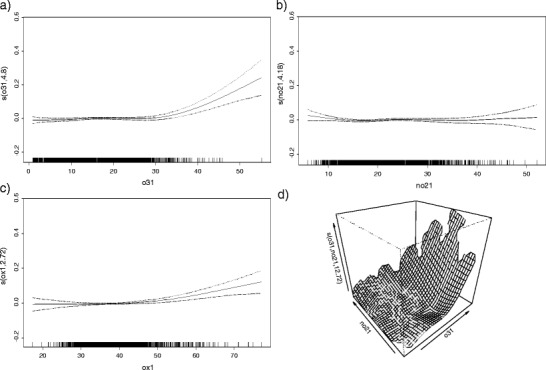
Concentration response functions for mean 24-h O_3_ (plot a), NO_2_ (plot b), O_x_ (plot c) and mortality in single-pollutant models and concentration response surface for for NO_2_ and O_3_ (plot d)

Figure 3 illustrates the concentration response functions for 24-h averages of O_3_, NO_2_ and O_x_ in single-pollutant models, and the concentration response surface for O_3_ and NO_2_ modelled jointly. A clear nonlinear relationship with mortality was observed for O_3_ (plot a) but not NO_2_ (plot b)—relationships that persisted upon mutual adjustment (plot d). The concentration-response function for O_x_ (plot c) was also suggestive of a nonlinear relationship.

## Discussion

### Summary of findings

Our time series analysis of daily mortality and daily concentrations of O_3_, NO_2_ and combined oxidant (O_x_) derived from the simple addition of O_3_ and NO_2_ revealed: (1) larger associations with mortality for 24-h concentrations of O_x_ than for O_3_ and NO_2_ individually; (2) stronger associations for both O_3_ and NO_2_ when analysed jointly in two-pollutant models compared to individual associations from single-pollutant models and (3) associations for O_x_ and for O_3_ and NO_2_ in two-pollutant models were larger for 24-h measures than for 1-h measures. In summer, and to a lesser extent during autumn months, ozone associations dominated the two-pollutant models; whereas during spring, both O_3_ and NO_2_ were positively associated with all-cause mortality. During these months, the O_x_ associations were similar to the O_3_ associations. No associations were observed during winter months for any of the pollutants.

### O_x_ results

Our descriptive analyses of daily concentrations of O_3_ and NO_2_ illustrate clearly the interrelation between the two pollutants and the two regions of O_x_ formation. Below hourly average concentrations of about 35 ppb, a typical tropospheric background ozone concentration (Monks et al. 2009), the ‘titration’ reaction (Eq. 1) dominates and ozone and NO_2_ are negatively correlated. Above this value, ozone tends to be produced photochemically and more complex reactions take place in addition to reactions (Eqs. 1–3) with the result that O_3_ and NO_2_ were positively correlated. The scatter plots and correlations statistics for the two pollutants by season illustrate both the productive nature of the relationship (e.g. during summer months) and the destructive nature (e.g. winter months). This strong interrelationship of the two pollutants with correlations that vary in direction and magnitude by season suggests that analyses of each pollutant separately will not characterise well the combined oxidative stress on the population from simultaneous exposure to the two pollutants. This observation appears to be supported by our finding that associations with mortality were larger for 24-h values of O_x_ than for O_3_ and NO_2_ from single-pollutant models.

### Single- and two-pollutant model results

A substantial epidemiological time series literature has demonstrated associations between O_3_ and NO_2_ and mortality in single-pollutant models (Anderson et al. 2007; WHO 2013). However, only a relatively small number of time series studies of all-age, all-cause mortality (all season) have considered O_3_ and NO_2_ jointly in two-pollutant models. In Europe, the APHEA-1 and -2 projects investigated both pollutants, initially in six cities (Touloumi et al. 1997) and then for 21 (O_3_) and 30 (NO_2_) European cities (Gryparis et al. 2004; Samoli et al. 2006). The initial investigation used 1-h measures of each pollutant and reported a small increase in both risk estimates when the pollutants were considered simultaneously in two-pollutant models compared with single-pollutant models. This contrasted with the summer only analysis in the later study where the summary effect estimate for 1-h ozone reduced by one third upon adjustment for 1-h NO_2_ concentrations (no adjusted NO_2_ coefficient reported). The analysis of 1-h NO_2_ in the APHEA-2 project reported a little change in the NO_2_ effect estimate upon adjustment for mean 8-h O_3_. Attenuation of the (mean 8-h) O_3_ coefficient (but not the NO_2_ coefficient) was observed in four Asian cities in the PAPA study (HEI 2010). Other multi-city studies to investigate these pollutants include studies in Spain (Saez et al. 2002), Canada (Burnett et al. 1998) and Australia (Simpson et al. 2005), each reporting little changes in the estimated effects on mortality of 1-h pollutant measures in single- and in two-pollutant models—findings consistent with our results for 1-h O_3_ and NO_2_—and suggestive of independent effects of the 1-h measures of the two pollutants. Our finding that the magnitude of the single-pollutant model results for 24-h measures of O_3_ and NO_2_ increase in two-pollutant models is, given the sparseness of the literature and the variety of averaging times used, difficult therefore to assess in relation to other studies.

### O_x_ result versus two-pollutant model results

In our study, we found that in the all-year and season-specific associations the O_x_ associations were generally comparable to the associations for O_3_ and NO_2_ estimated from two-pollutant models. The importance of multi-pollutant approaches for the evaluation of health risks associated with exposures to air pollution has been widely recognised (Johns 2011). Jerret and colleagues noted that (in their recent cohort study of mortality) ‘both pollutants need to be in the model for correct inference on either’ (Jerret et al. 2013). However, this issue is not straightforward since different amounts of measurement error in the pollutant concentrations can cause biases that require careful adjustment (Zeka and Schwartz 2004). Multi-pollutant models also rely upon other assumptions regarding the linearity of the concentration response function and a lack of seasonal differences in confounding amongst others further complicating their interpretation (Kim et al. 2007). These concerns pose policy makers with problems of interpretation of model results. Based upon atmospheric chemistry alone, there is a strong, a priori, reason for considering O_3_ and NO_2_ together in epidemiological studies. From a policy perspective, the use of a single metric for health impact assessment is also appealing as it simplifies health impact calculations and avoids any possible double counting, although the monitoring requirements remain unchanged.

### Max 1-h versus 24-h results

Few studies have investigated both 1-h and 24-h O_3_ and NO_2_ and daily, all-cause mortality within the same dataset. Burnett and colleagues studied both pollutants in 12 Canadian cities and concluded that the daily 1-h concentrations for NO_2_ displayed a weaker association with daily mortality compared to the daily average values but that ‘the daily average value of O_3_ also displayed a weaker association with mortality than did the daily 1-h maximum concentration’ (Burnett et al. 2004). Studies from North America have tended to focus on 24-h measures of ozone (e.g. analysis of 95 US cities by Bell et al. (2005)), whereas multi-city studies in Europe have used maximum 1- or 8-h measures (Gryparis et al. 2004). In Asia, the large, multi-city PAPA study used 8-h measures (HEI 2010). Our finding of larger associations with 24-h measures compared to 1-h measures in single-pollutant models is therefore new. It suggests that, in our data at least, the longer exposure periods for both pollutants together better characterises daily oxidant exposure although we note that in our study, as in general, the daily measures were highly correlated (*r* = 0.9 for both O_3_ and NO_2_).

### Mechanism of effects

In deciding on an appropriate model or metric to account for the atmospheric chemistry which interchanges O_3_ and NO_2_, a consideration of the mechanism of effects is relevant. If both pollutants exert their adverse effects via oxidative stress, then adding them to form O_x_ seems particularly relevant. If different mechanisms apply, there is still an argument based on the fundamental atmospheric chemistry for incorporating both O_3_ and NO_2_ in the same study, but this could involve either the use of O_x_ or a traditional two-pollutant model. As well as acting through oxidative stress, it is possible that NO_2_ could exert its effects also through protein nitration (Matalon et al. 2009). In this case, if O_x_ is calculated as a sum of O_3_ and NO_2_ weighted by their oxidative potentials, then the effects of NO_2_ could possibly be underestimated since O_3_ has a much larger oxidation potential than does NO_2_. This is discussed further in the next section.

It also needs to be borne in mind that the simple chemistry in Eqs. 1–3 describes the O_3_/NO_x_ system during much of the year, but during periods of more intense photochemical activity, in so-called ‘smog’ episodes, more complex chemistry occurs involving reactions of NO_x_ and volatile organic compounds (VOCs). These reactions produce intermediates (for example peroxy radicals) and products which could potentially lead to adverse health effects—peroxyacetyl nitrate (PAN) for example is known to exert adverse effects on health. However, the problem for epidemiological studies is that these species are generally difficult to measure so that no routine measurements are currently available.

A further consideration is the fact that in many urban areas the main source of NO_x_ and hence NO_2_ is road traffic, so that NO_2_ is often closely correlated with particulate matter (PM) and particularly measures of primary PM such as ultrafine particles and elemental and/or black carbon. Further work will be necessary to explore the combined effects of O_3_, NO_2_ and PM.

### Alternative weighting for O_x_

As noted in the previous section, in terms of chemical redox potentials, O_3_ might be expected to be the more powerful oxidant with a redox potential of 2.075 V compared with a value of 1.07 V for NO_2_ (Bratsch 1989). As a measure of combined oxidant, a weighted average of O_3_ and NO_2_ may therefore better represent the total impact of the two pollutants on health if oxidative stress is the main mechanism operating for both pollutants. As noted above, however, NO_2_ may also act via other mechanisms such as protein nitration, so in a sensitivity analysis we recalculated the O_x_ metrics using the above weighting. For all-year mortality, the weighted O_x_ metric was slightly larger than the unweighted metric but its precision was poorer (data not shown).

### Thresholds

Another issue of relevance is the question of thresholds—concentrations below which there are very few or undetectable health effects. Here, the atmospheric chemistry of the O_3_/NO_x_ system could clearly be important. As noted above, typical average tropospheric background levels of O_3_ are around 35 ppb, but lower concentrations of O_3_ occur in urban areas (particularly when O_3_ ‘smog’ episodes are not occurring) because of the reaction of O_3_ with NO, when the O_3_ is converted to NO_2_. Investigating the existence or otherwise of a threshold for O_3_ (and possibly for NO_2_ although less work has been done to date on this issue) should therefore account in some way for the atmospheric chemistry inherent in the system and should recognise that, particularly in populated urban areas, low ozone concentrations occur because much, or even all, of the ozone is converted to NO_2_. Single-pollutant studies of ozone which attempt to identify thresholds without taking the NO_2_ interaction into account may therefore give misleading results. Our preliminary analysis of this issue suggests that the evidence for nonlinearity on the 24-h O_3_ mortality concentration response relationship reported in this study, and previously in a longer time series for London (Atkinson et al. 2012), remains unaffected by the inclusion of NO_2_ in the model. This issue has received little attention even in large and influential multi-city studies (e.g. Bell et al. 2006).

### Conclusion

Our study has demonstrated that the strong interrelationship between daily concentrations of O_3_ and NO_2_ suggests that analysis of each pollutant separately does not capture adequately the combined effects on the population from simultaneous exposure to the two interacting pollutants. Based upon atmospheric chemistry alone, there is a strong, a priori, reason for considering O_3_ and NO_2_ together in epidemiological studies, rather than either of the two pollutants separately in single-pollutant models. This paper has compared two approaches to this, using O_x_, defined as O_3_ + NO_2_, as a single metric and also using O_3_ and NO_2_ together in two-pollutant models. Our work has shown that single-pollutant models of either O_3_ or NO_2_ can potentially give misleading results, and some form of combination of the two pollutants needs to be considered in epidemiological studies, either as O_x_ or in a two-pollutant model. Furthermore, we suggest that a single metric O_x_ has advantages over the traditional two-pollutant model approach as it avoids many of the statistical issues associated with such approaches and also simplifies health impact calculations. Also, from a policy perspective, the use of a single metric for health impact assessment is appealing, although the monitoring requirements would remain unchanged. Further work is required to confirm the findings from this study in other cities and countries; other health outcomes and diseases and the potential confounding of these relationships by fine particles. At the very least interactions between ozone and NO_2_ should be considered carefully in future epidemiological studies and in policy reviews.

## Electronic supplementary material

Below is the link to the electronic supplementary material.

ESM 1 (DOCX 175 kb)
