# Supplementary material for: Associations between daily mortality in London and combined oxidant capacity, ozone and nitrogen dioxide
Source: Air Qual Atmos Health. 2014 Mar 22;7(4):407–14. doi: 10.1007/s11869-014-0249-8 (PMC4239710; doi:10.1007/s11869-014-0249-8)
Supplement: Supplementary file 1 — (DOCX 175 kb) [file 11869_2014_249_MOESM1_ESM.docx]

SUPPLEMENTARY INFORMATION FOR:

Associations between daily mortality in London and combined oxidant capacity, ozone and nitrogen dioxide

Williams M.L.^1^, Atkinson R.W.^2^, Anderson H.R.^1,2^, Kelly F.J.^1^

1 – MRC-PHE Centre for Environment and Health, Kings College London

2 – Division of Population Health Sciences and Education and MRC-PHE Centre for Environment and Health, St George’s, University of London, London

Address for correspondence

Professor Martin Williams

MRC-PHE Centre for Environment and Health,

Kings College London

Room 4.129 Franklin Wilkins Building

150 Stamford Street

London SE1 9NH

UK

Email: [martin.williams@kcl.ac.uk](mailto:martin.williams@kcl.ac.uk)

Tel.: +44 7765 203 627 or +44 207 848 3844

Keywords: Time series, mortality, oxidants, ozone, nitrogen dioxide

Table S1 Summary statistics for daily maximum 1-hr and mean 24-hr concentrations of ozone (O_3_), nitrogen dioxide (NO_2_) and combined oxidant (O_x_) by season

| Season/  Measure | Metric  (ppb) | Min^1^ | Q1^2^ | Med^3^ | Q3^4^ | Max^5^ | IQR^6^ |
| --- | --- | --- | --- | --- | --- | --- | --- |
|  |  |  |  |  |  |  |  |
| Dec-Feb |  |  |  |  |  |  |  |
| 1-hr | O_3_ | 1.7 | 15.9 | 24.2 | 30.6 | 45.2 | 14.7 |
|  | NO_2_ | 9.0 | 29.9 | 35.8 | 42.2 | 71.9 | 12.3 |
|  | O_x_ | 30.5 | 41.5 | 44.7 | 48.3 | 77.3 | 6.9 |
|  |  |  |  |  |  |  |  |
| 24-hr | O_3_ | 0.9 | 5.9 | 11.7 | 18.3 | 31.7 | 12.5 |
|  | NO_2_ | 5.9 | 18.0 | 22.8 | 27.8 | 46.7 | 9.8 |
|  | O_x_ | 25.2 | 34.4 | 37.1 | 39.7 | 52.7 | 5.3 |
|  |  |  |  |  |  |  |  |
| Mar-May |  |  |  |  |  |  |  |
| 1-hr | O_3_ | 7.1 | 31.0 | 36.3 | 40.4 | 68.2 | 9.5 |
|  | NO_2_ | 11.1 | 28.9 | 35.6 | 42.0 | 78.0 | 13.1 |
|  | O_x_ | 31.2 | 48.7 | 52.1 | 56.8 | 94.7 | 8.0 |
|  |  |  |  |  |  |  |  |
| 24-hr | O_3_ | 2.2 | 16.2 | 21.1 | 26.6 | 44.6 | 10.4 |
|  | NO_2_ | 6.6 | 16.6 | 20.8 | 25.2 | 47.4 | 8.6 |
|  | O_x_ | 21.4 | 40.3 | 42.9 | 46.2 | 67.6 | 5.9 |
|  |  |  |  |  |  |  |  |
| Jun-Aug |  |  |  |  |  |  |  |
| 1-hr | O_3_ | 12.9 | 28.6 | 33.8 | 41.0 | 103.9 | 12.4 |
|  | NO_2_ | 12.2 | 24.1 | 30.2 | 35.7 | 66.2 | 11.6 |
|  | O_x_ | 25.0 | 42.8 | 48.7 | 57.3 | 123.5 | 14.5 |
|  |  |  |  |  |  |  |  |
| 24-hr | O_3_ | 3.9 | 15.2 | 19.0 | 23.2 | 55.2 | 8.0 |
|  | NO_2_ | 8.6 | 14.0 | 17.2 | 20.8 | 42.6 | 6.8 |
|  | O_x_ | 17.4 | 33.0 | 37.0 | 42.1 | 77.1 | 9.1 |
|  |  |  |  |  |  |  |  |
| Sep-Nov |  |  |  |  |  |  |  |
| 1-hr | O_3_ | 2.4 | 19.5 | 25.1 | 29.9 | 73.2 | 10.4 |
|  | NO_2_ | 12.0 | 29.6 | 35.1 | 40.7 | 81.3 | 11.2 |
|  | O_x_ | 28.4 | 41.4 | 44.5 | 49.1 | 94.5 | 7.7 |
|  |  |  |  |  |  |  |  |
| 24-hr | O_3_ | 1.1 | 7.5 | 11.8 | 16.6 | 33.6 | 9.1 |
|  | NO_2_ | 7.4 | 17.8 | 21.8 | 26.2 | 52.0 | 8.4 |
|  | O_x_ | 23.6 | 33.2 | 35.9 | 38.6 | 71.1 | 5.4 |
|  |  |  |  |  |  |  |  |

Notes: 1- Minimum; 2 – 25^th^ percentile; 3 – Median; 4 – 75^th^ percentile; 5 – Maximum; 6 – Iterquartile range

Table S2 Spearman rank correlations coefficients between daily maximum 1-hr and mean 24-hr concentrations of ozone (O_3_), nitrogen dioxide (NO_2_) and combined oxidant (O_x_) for months December to February

| Pollutant | O_3_ | NO_2_ | O_X_ | O_3_ | NO_2_ | O_X_ |
| --- | --- | --- | --- | --- | --- | --- |
|  |  | | |  | | |
|  | 1-hr | | | 24-hr | | |
| 1-hr |  |  |  |  |  |  |
| O_3_ | 1.00 |  |  |  |  |  |
| NO_2_ | -0.50 | 1.00 |  |  |  |  |
| O_X_ | -0.12 | 0.76 | 1.00 |  |  |  |
|  |  |  |  |  |  |  |
| 24-hr |  |  |  |  |  |  |
| O_3_ | 0.91 | -0.65 | -0.18 | 1.00 |  |  |
| NO_2_ | -0.73 | 0.89 | 0.61 | -0.84 | 1.00 |  |
| O_X_ | 0.28 | 0.44 | 0.82 | 0.24 | 0.29 | 1.00 |
|  |  |  |  |  |  |  |

Table S3 Spearman rank correlations coefficients between daily maximum 1-hr and mean 24-hr concentrations of ozone (O_3_), nitrogen dioxide (NO_2_) and combined oxidant (O_x_) for months March to May

| Pollutant | O_3_ | NO_2_ | O_X_ | O_3_ | NO_2_ | O_X_ |
| --- | --- | --- | --- | --- | --- | --- |
|  |  | | |  | | |
|  | 1-hr | | | 24-hr | | |
| 1-hr |  |  |  |  |  |  |
| O_3_ | 1.00 |  |  |  |  |  |
| NO_2_ | -0.11 | 1.00 |  |  |  |  |
| O_X_ | 0.60 | 0.53 | 1.00 |  |  |  |
|  |  |  |  |  |  |  |
| 24-hr |  |  |  |  |  |  |
| O_3_ | 0.77 | -0.58 | 0.17 | 1.00 |  |  |
| NO_2_ | -0.26 | 0.90 | 0.46 | -0.71 | 1.00 |  |
| O_X_ | 0.66 | 0.39 | 0.88 | 0.39 | 0.33 | 1.00 |
|  |  |  |  |  |  |  |

Table S3 Spearman rank correlations coefficients between daily maximum 1-hr and mean 24-hr concentrations of ozone (O_3_), nitrogen dioxide (NO_2_) and combined oxidant (O_3_) for months June to August

| Pollutant | O_3_ | NO_2_ | O_X_ | O_3_ | NO_2_ | O_X_ |
| --- | --- | --- | --- | --- | --- | --- |
|  |  | | |  | | |
|  | 1-hr | | | 24-hr | | |
| 1-hr |  |  |  |  |  |  |
| O_3_ | 1.00 |  |  |  |  |  |
| NO_2_ | 0.51 | 1.00 |  |  |  |  |
| O_X_ | 0.93 | 0.70 | 1.00 |  |  |  |
|  |  |  |  |  |  |  |
| 24-hr |  |  |  |  |  |  |
| O_3_ | 0.85 | 0.17 | 0.72 | 1.00 |  |  |
| NO_2_ | 0.45 | 0.92 | 0.67 | 0.08 | 1.00 |  |
| O_X_ | 0.90 | 0.67 | 0.95 | 0.80 | 0.63 | 1.00 |
|  |  |  |  |  |  |  |

Table S4 Spearman rank correlations coefficients between daily maximum 1-hr and mean 24-hr concentrations of ozone (O_3_), nitrogen dioxide (NO_2_) and combined oxidant (O_x_) for months September to November

| Pollutant | O_3_ | NO_2_ | O_X_ | O_3_ | NO_2_ | O_X_ |
| --- | --- | --- | --- | --- | --- | --- |
|  |  | | |  | | |
|  | 1-hr | | | 24-hr | | |
| 1-hr |  |  |  |  |  |  |
| O_3_ | 1.00 |  |  |  |  |  |
| NO_2_ | -0.13 | 1.00 |  |  |  |  |
| O_X_ | 0.43 | 0.72 | 1.00 |  |  |  |
|  |  |  |  |  |  |  |
| 24-hr |  |  |  |  |  |  |
| O_3_ | 0.85 | -0.44 | 0.16 | 1.00 |  |  |
| NO_2_ | -0.33 | 0.91 | 0.59 | -0.62 | 1.00 |  |
| O_X_ | 0.57 | 0.57 | 0.91 | 0.39 | 0.44 | 1.00 |
|  |  |  |  |  |  |  |

Table S6 Regression coefficients and standard errors from Poisson regression models for all-year and seasonal, single pollutant models for ozone (O_3_), nitrogen dioxide (NO_2_) and combined oxidant (O_x_) and for two-pollutant models for O_3_ and NO_2_ for maximum 1-hr and mean 24-hr pollutant concentrations lagged 1 day for daily mortality in London

| Metric | All-Year | |  | Dec-Feb | |  | Mar-May | |  | Jun-Aug | |  | Sept-Nov | |
| --- | --- | --- | --- | --- | --- | --- | --- | --- | --- | --- | --- | --- | --- | --- |
|  | b^1^ | se^2^ |  | b^1^ | se^2^ |  | b^1^ | se^2^ |  | b^1^ | se^2^ |  | b^1^ | se^2^ |
|  |  |  |  |  |  |  |  |  |  |  |  |  |  |  |
| 1-hr |  |  |  |  |  |  |  |  |  |  |  |  |  |  |
| Single-pollutant | |  |  |  |  |  |  |  |  |  |  |  |  |  |
| O_3_ | 0.676 | 0.275 |  | -0.357 | 0.389 |  | 1.389 | 0.368 |  | 1.116 | 0.365 |  | 0.759 | 0.383 |
| NO_2_ | 0.244 | 0.238 |  | -0.296 | 0.301 |  | 1.055 | 0.316 |  | 0.151 | 0.374 |  | 0.007 | 0.315 |
| O_x_ | 0.416 | 0.269 |  | -0.292 | 0.327 |  | 0.964 | 0.316 |  | 0.645 | 0.320 |  | 0.225 | 0.314 |
|  |  |  |  |  |  |  |  |  |  |  |  |  |  |  |
| Two-pollutant | |  |  |  |  |  |  |  |  |  |  |  |  |  |
| O_3_ | 0.729 | 0.277 |  | -0.065 | 0.410 |  | 0.843 | 0.421 |  | 1.621 | 0.481 |  | 0.750 | 0.433 |
| NO_2_ | 0.331 | 0.240 |  | 0.017 | 0.314 |  | 0.989 | 0.367 |  | -0.630 | 0.522 |  | 0.058 | 0.348 |
|  |  |  |  |  |  |  |  |  |  |  |  |  |  |  |
| 24-hr |  |  |  |  |  |  |  |  |  |  |  |  |  |  |
| Single pollutant | |  |  |  |  |  |  |  |  |  |  |  |  |  |
| O_3_ | 0.871 | 0.339 |  | -0.604 | 0.525 |  | 1.490 | 0.468 |  | 1.950 | 0.561 |  | 1.145 | 0.568 |
| NO_2_ | 0.002 | 0.358 |  | -0.701 | 0.440 |  | 1.400 | 0.493 |  | -0.253 | 0.619 |  | -0.403 | 0.483 |
| O_x_ | 1.296 | 0.432 |  | 0.321 | 0.503 |  | 1.908 | 0.480 |  | 1.537 | 0.487 |  | 0.972 | 0.485 |
|  |  |  |  |  |  |  |  |  |  |  |  |  |  |  |
| Two-pollutant | |  |  |  |  |  |  |  |  |  |  |  |  |  |
| O_3_ | 1.541 | 0.450 |  | 0.379 | 0.635 |  | 1.786 | 0.551 |  | 3.171 | 0.690 |  | 1.570 | 0.643 |
| NO_2_ | 1.072 | 0.475 |  | 0.433 | 0.559 |  | 2.434 | 0.608 |  | -0.256 | 0.753 |  | 0.599 | 0.560 |
|  |  |  |  |  |  |  |  |  |  |  |  |  |  |  |

Note: Regression coefficient (1) and standard error (2) x1000 per ppb

Figure S1 Hourly average concentrations (µg/m^3^) of ozone (O_3_), nitrogen dioxide (NO_2_) and combined oxidant (Ox)and ozone/oxidant ratio during the heatwave in London, August 1^st^-13^th^ 2003.


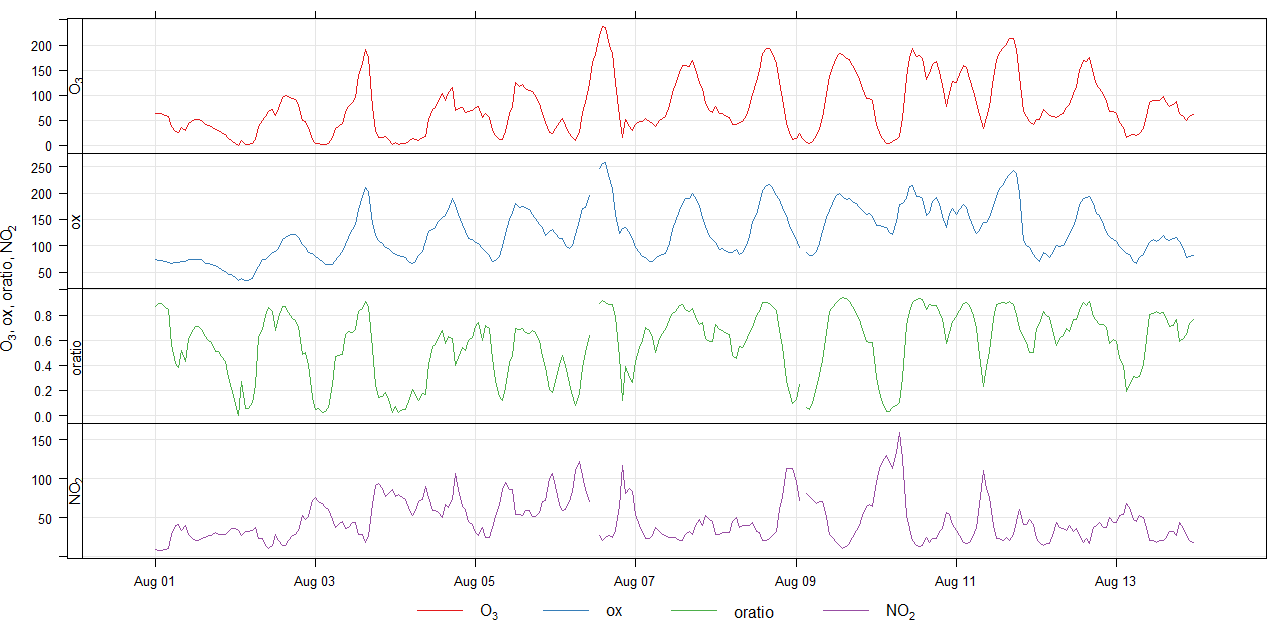


Figure S2 Scatter plots of daily maximum 1-hr and mean 24-hr concentrations of nitrogen dioxide and ozone in London between 1^st^ January 2000 and 31^st^ December 2005 for months December to February

1. 1-hr

1. 24-hr

Figure S3 Scatter plots of daily maximum 1-hr and mean 24-hr concentrations of nitrogen dioxide and ozone in London between 1^st^ January 2000 and 31^st^ December 2005 for months March to May

1. 1-hr

1. 24-hr

Figure S4 Scatter plot of daily maximum 1-hr and mean 24-hr concentrations of nitrogen dioxide and ozone in London between 1^st^ January 2000 and 31^st^ December 2005 for months June to August

1. 1-hr

1. 24-hr

Figure S5 Scatter plot of daily maximum 1-hr and mean 24-hr concentrations of nitrogen dioxide and ozone in London between 1^st^ January 2000 and 31^st^ December 2005 for months September to November

1. 1-hr

1. 24-hr

Figure S6 Percentage changes (95% confidence intervals) in mortality associated with lag 1 day measures of maximum 1-hr and mean 24-hr concentrations of ozone (O_3_), nitrogen dioxide (NO_2_) and combined oxidant (O_x_) in single pollutant models and ozone and nitrogen dioxide in two-pollutant models during December to February

1. Per 10 ppb
2. Per interquartile range

Figure S7 Percentage changes (95% confidence intervals) in mortality associated with lag 1 day measures of maximum 1-hr and mean 24-hr concentrations of ozone (O_3_), nitrogen dioxide (NO_2_) and combined oxidant (O_x_) in single pollutant models and ozone and nitrogen dioxide in two-pollutant models during March to May

1. Per 10 ppb
2. Per interquartile range

Figure S8 Percentage changes (95% confidence intervals) in mortality associated with lag 1 day measures of maximum 1-hr and mean 24-hr concentrations of ozone (O_3_), nitrogen dioxide (NO_2_) and combined oxidant (O_x_) in single pollutant models and ozone and nitrogen dioxide in two-pollutant models during June to August

1. Per 10 ppb
2. Per interquartile range

Figure S9 Percentage changes (95% confidence intervals) in mortality associated with lag 1 day measures of maximum 1-hr and mean 24-hr concentrations of ozone (O_3_), nitrogen dioxide (NO_2_) and combined oxidant (O_x_) in single pollutant models and ozone and nitrogen dioxide in two-pollutant models during September to November

1. Per 10 ppb
2. Per interquartile range
